# Transcatheter arterial embolization for acute lower gastrointestinal bleeding using imipenem/cilastatin: a single-center retrospective study

**DOI:** 10.1186/s42155-023-00359-w

**Published:** 2023-03-10

**Authors:** Sakiko Hiraki, Fumie Sato, Masaya Osugi, Yoshiya Watanabe, Yoshiaki Ichinose

**Affiliations:** Department of Radiology, National Hospital Organization Disaster Medical Center, 3256, Midoricho, Tokyo, Tachikawa 190-0014 Japan

**Keywords:** Imipenem/cilastatin, IPM/CS, Lower gastrointestinal bleeding, Transcatheter arterial embolization

## Abstract

**Background:**

Transcatheter arterial embolization (TAE) is a standard treatment for acute lower gastrointestinal bleeding (LGIB) in situations where endoscopic approaches are impossible or ineffective. Various embolic materials, such as metallic coils and N-butyl cyanoacrylate, are used. This study aimed to evaluate the clinical outcomes of an imipenem/cilastatin (IPM/CS) mixture as an embolic agent in TAE for acute LGIB.

**Results:**

Twelve patients (mean age, 67 years) with LGIB treated with TAE using IPM/CS were retrospectively evaluated between February 2014 and September 2022. All patients showed evidence of extravasation on computed tomography and 50% (6/12) also showed evidence on angiography. The technical success rate for TAE in this study was 100%, including in patients who showed active extravasation on angiography. The clinical success rate was 83.3% (10/12), with two patients experiencing rebleeding within 24 h after the procedure. No ischemic complications were observed, and no bleeding episodes or other complications were reported during the follow-up period.

**Conclusions:**

This study revealed that using IPM/CS as an embolic agent in TAE for acute LGIB may be safe and effective, even in cases of active bleeding.

## Background

Transcatheter arterial embolization (TAE) is a standard treatment for lower gastrointestinal bleeding (LGIB) in situations where endoscopic approaches are impossible or ineffective. Various embolic materials, such as metallic coils and N-butyl cyanoacrylate (NBCA), are used (Nagata et al. 2019; Oakland et al. 2019; Lee et al. 2022; Chevallier et al. 2021; Funaki et al. 2001a; Kinoshita et al. 2021). However, in some cases, superselective embolization using coils may be impossible or unfeasible. In these situations, the use of NBCA may be considered, but it is associated with the risk of ischemic complications in non-superselective situations (Ikoma et al. 2010; Kodani et al. 2016). In 2013, the efficacy of imipenem/cilastatin (IPM/CS) as an embolization agent for treating acute LGIB was reported (Woodhams et al. 2013).

This study aimed to determine the effectiveness of a mixture of IPM/CS as an alternative embolization agent in TAE for acute LGIB in situations where superselective embolization using microcoils is impossible or unfeasible. In addition, this study also aimed to evaluate the clinical outcomes of using the IPM/CS mixture as an embolic agent in TAE for acute LGIB.

## Methods

### Patient population

This single-center retrospective study evaluated the outcomes of TAE using IPM/CS as an embolic agent for acute LGIB. A total of 47 patients underwent angiography to evaluate acute LGIB from February 2014 to September 2022, including 12 patients (nine males and three females; age range, 37–93 years; mean age, 67 years) treated with TAE using IPM/CS as an embolic agent. All patients suspected of having LGIB underwent dynamic contrast-enhanced multi-detector computed tomography (MDCT) to identify the extravasation's presence and location. Only one patient underwent trans-arterial contrast-enhanced MDCT with a catheter in the superior mesenteric artery (SMA). Trans-arterial contrast-enhanced MDCT imaging is generally not performed at our institution, but this patient specifically underwent trans-arterial contrast-enhanced MDCT because he had a history of chronic idiopathic pseudo-intestinal obstruction that precluded endoscopy, although small bowel bleeding was strongly suspected. If active extravasation was identified on the MDCT scan and catheter access was possible, TAE was first performed to stop the bleeding followed by endoscopy. All patients were treated at the National Hospital Organization Disaster Medical Center in Tokyo, Japan, and provided written informed consent for the procedure, which included information regarding the potential risks and benefits.

### TAE methods and techniques

Angiography was performed by one or two of our radiologists: one of the three attending radiologists in our unit who had 5 years of experience in this field or fellows under the close supervision of a radiologist. IPM/CS as an embolic agent is not typically covered by insurance in Japan; therefore, we obtained approval from the institutional review board before using this treatment.

All procedures were performed using an angiographic system (Allura Xper FD 20/10 or Azurion 7 M20, Philips Healthcare, Best, The Netherlands) under local anesthesia administered through the femoral artery using a 4- or 5-French (Fr) sheath (Medikit Super Sheath, Medikit, Tokyo, Japan; Radiforcus Introducer IIH, Terumo, Tokyo, Japan). A 4-Fr angiographic catheter shepherd hook-type catheter (SHK or SHA, Medikit, Tokyo, Japan) or 5-Fr guiding catheter Cobra (C1, Medikit, Tokyo, Japan) was injected with 2–4 mL/s for 5 s via injector into the SMA or inferior mesenteric artery (IMA), and diagnostic angiography was performed to identify the bleeding site. Even if the bleeding site could not be identified using SMA or IMA arteriography, arteriography using the SMA or IMA branches (right colic, middle colic, ileocolic, and sigmoid arteries) or selective angiography using the vasa recta or marginal artery was performed using 1.9- to 2.9-Fr microcatheters (BISHOP HF, PIOLAX, Kanagawa, Japan; LEONIS Mova HF, Sumitomo Bakelite, Tokyo, Japan; Carnelian® MARVEL Non-Taper or Carnelian® MARVEL S, Tokai Medical Products, Aichi, Japan). The injection from the microcatheter varies from case to case and can be performed by hand or by the injector. No case underwent cone-beam CT (CBCT).

Our team had a standard approach for managing cases of acute LGIB using TAE. Our priority was to identify the bleeding vessel using contrast-enhanced MDCT and selective angiography, using the vasa recta or marginal artery; and, if possible, stopping the bleeding using superselective embolization with microcoils. If this was impossible or unfeasible, the team would use IPM/CS as an alternative embolic material. Based on this management technique, the final decision regarding which embolization agent to use and whether to perform the embolization procedure was at the operators’ discretion, based on their clinical judgment.

First, we prepared an intraarterial injection of a mixture of IPM/CS (0.5 g) and nonionic contrast medium (5 mL). Then, the mixture was drawn into a syringe and gently pumped approximately 10 times to ensure it was mixed properly. The mixture was mainly injected through a high-flow microcatheter or a microcatheter placed in a marginal artery near the bleeding site until the extravasation disappeared or the blood flow in the bleeding site and the surrounding marginal artery became stagnant. In some cases, it was necessary to inject the mixture until the IPM/CS was distributed in a cast-like fashion from the level of the marginal arteries to achieve this effect.

After the embolization procedure was completed, angiography was performed to confirm the technical success of the procedure and check for any other possible sources of bleeding.

### Assessment

Technical success was defined as the success of the angiographic procedure based on the disappearance of extravasation or blood flow in the target artery. Clinical success was defined as the cessation of bleeding without needing additional hemostatic treatment within 7 days. Early recurrent bleeding was defined as bleeding from the treated area within 3 days after TAE. Complications from embolization procedures can be divided into two categories: "non-specific" and "specific". Non-specific complications are those that are due to the angiographic procedure itself, such as bleeding, infection, or allergic reactions to the materials used. Specific complications refer to those that are specific to the area being embolized, such as intestinal ischemia in the case of intestinal embolization.

## Results

Patients’ backgrounds are shown in Table 1. This study included 12 patients, of whom six were taking antithrombotic medications. The etiology of bleeding was colonic diverticulosis in eight patients and bleeding in the small bowel due to inflammatory, ulcerative, cancerous, or unknown causes in the remaining four patients. All but one patient had evidence of extravasation on the MDCT scan. This patient had micro-extravasation on a trans-arterial contrast-enhanced MDCT with a catheter in the SMA.

**Table 1:** Patients’ backgrounds

	**n (%)**
Sex	
Male	9 (75%)
Female	3 (25%)
Age (range: 37–93 years)	
> 60	7 (58%)
≦60	5 (42%)
Antithrombotic medications	
No	6 (50%)
Yes	6 (50%)
Etiology of bleeding	
colonic diverticulosis	8 (68%)
inflammatory	1 (8%)
ulcerative	1 (8%)
cancerous	1 (8%)
unknown	1 (8%)
Site of bleeding	
small bowel	4 (34%)
Right-sided colon	5 (42%)
Left-sided colon	1 (8%)
Transverse colon	1 (8%)
Rectosigmoid	1 (8%)

The summary of TAE outcomes is shown in Table 2. Extravasation appeared on angiography in 50% of the patients (6/12), most of which occurred in the SMA, which supplies blood to the small intestine and right-sided colon. IPM/CS was used as an embolization agent for six patients because of the disappearance of extravasation on angiography and difficulty in catheterizing the bleeding vasa recta for the remaining six patients. In all patients, the microcatheter was located at the marginal artery of the bleeding site during embolization.

**Table 2:** Angiographic findings

	**n(%)**
Angiographic extravasation	
No	6 (50%)
Yes	6 (50%)
Bleeding territory	
SMA	10 (83%)
IMA	2 (17%)
The reason why the IPM/CS was chosen	
disappearance of extravasation on angiography	6 (50%)
difficulty in catheterizing the bleeding vasa recta	6 (50%)

**Fig. 1:**
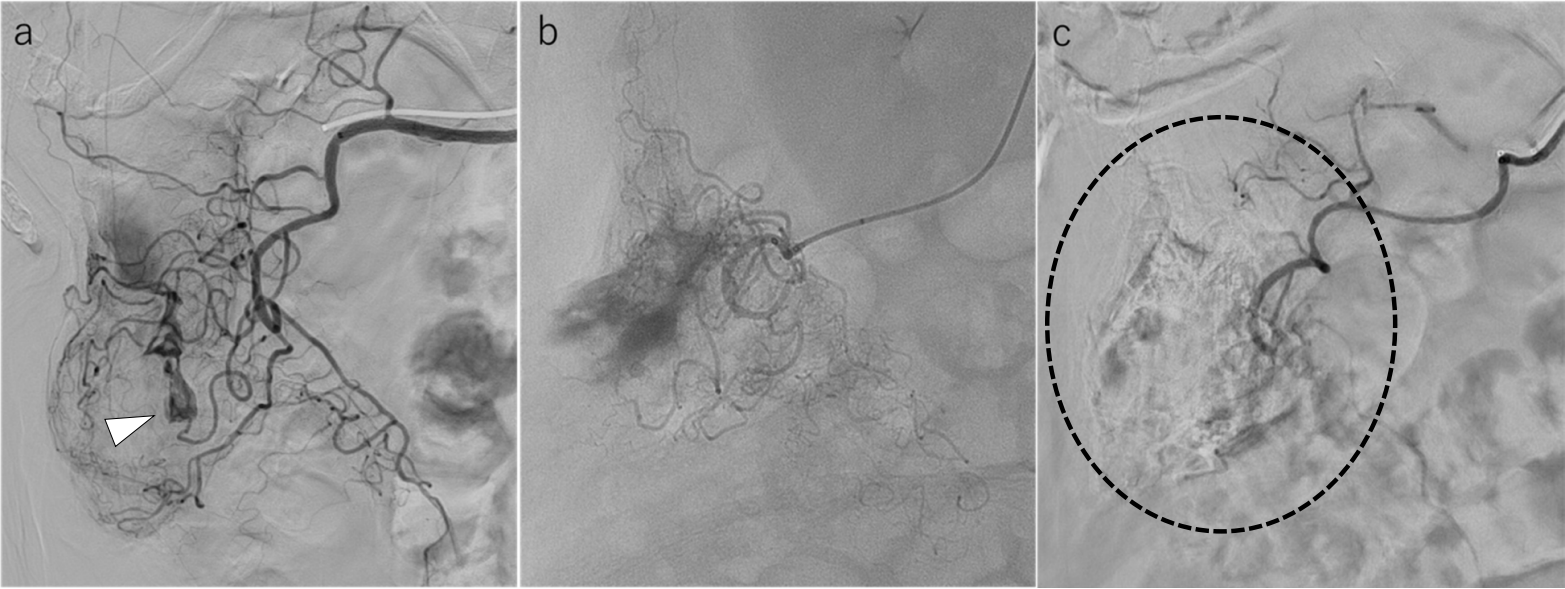
A 93-year-old woman with ascending colonic hemorrhage due to colonic diverticulosis. **a** Selective angiography of the ileocolic artery. Clear extravasation appears (white arrowhead); however, the catheterization of the bleeding vasa recta is not achieved. **b** Single shot while embolizing with the IPM/CS mixture; the microcatheter during embolization is located at the marginal artery of the bleeding site. We injected the IPM/CS mixture to distribute not only to the bleeding vasa recta but also to the surrounding vasa recta and the marginal artery. **c** Selective angiography of the ileocolic artery after the embolization using the IPM/CS mixture. The clear extravasation disappears and the blood flow to the bleeding straight artery and surrounding straight arteries is blocked (wave circled)

**Fig. 2:**
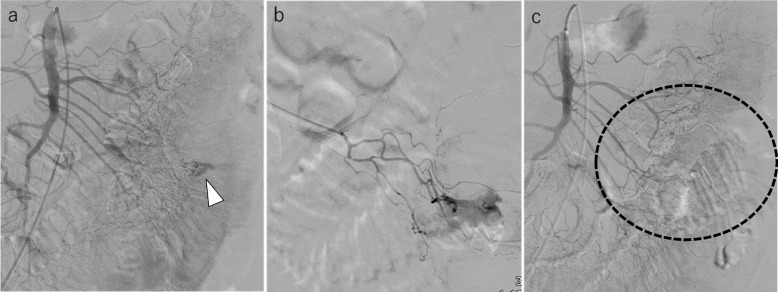
A 58-year-old man with a small bowel hemorrhage. **a** SMA angiography reveals active extravasation at the third branch of the jejunal artery (white arrowhead). **b** Selective angiography of the anastomotic arcade of the third branch reveals active extravasation. We attempted catheterization of the straight artery; however, this was difficult. Therefore, we performed embolization with an IPM/CS mixture using a microcatheter positioned at the anastomotic arcade. **c** Angiography of the SMA after embolization using an IPM/CS mixture reveals that the active extravasation has disappeared (wave circle)

The technical success rate for TAE in this study was 100% (12/12), which included patients in whom active extravasation was identified on angiography (Figs. 1 and 2). The clinical success rate was 83.3% (10/12), with two patients experiencing rebleeding within 24 h after the procedure. Both patients had bleeding from the same site; one underwent a right hemicolectomy and the other underwent endoscopic hemostasis. The physical examination and laboratory data showed no complications associated with the TAE technique or ischemia. In addition, no bleeding episodes or other complications were observed clinically or on MDCT images during the follow-up period. The mean follow-up period was 7.5 months (0.5–60 months).

## Discussion

IPM/CS is an antibiotic and was used as an embolic agent for chemoembolization in a 1999 animal study (Aihara 1999). Previous studies have reported that IPM/CS may have unique characteristics that make it a useful embolic agent, including the production of particles predominantly < 60 µm in size and the relatively short embolic effect of < 48 h (Aihara 1999; Yamada et al. 2021). In 2013, its efficacy as an embolization agent for treating acute LGIB was reported (Woodhams et al. 2013). Since 2014, IPM/CS has been used as an alternative embolic agent by our team in cases where superselective embolization using microcoils was difficult to achieve.

In the past report, IPM/CS was introduced from the proximal site of the artery to cover the tumor staining, and no major ischemic complication was suggested based on physical examinations and laboratory data (Woodhams et al. 2013). In our series, two patients who underwent selective embolization at the level of the vasa recta experienced rebleeding within 24 h. In contrast, others who underwent proximal embolization underwent injection through a high-flow microcatheter placed in a marginal artery near the bleeding site until the blood flow of the bleeding vasa recta and surrounding vasa recta became stagnant and there was no rebleeding or ischemic complications. Proximal embolization using a gelatin sponge, microcoil, or NBCA is not recommended because of the possibility of ischemic complications (Funaki et al. 2001a; Kinoshita et al. 2021; Ikoma et al. 2010; Kodani et al. 2016). However, proximal embolization with IPM/CS seems necessary to avoid rebleeding.

The safety of proximal embolization using an IPM/CS mixture has been reported in the human intestine and in treating tendinopathy and enthesopathy (Woodhams et al. 2013; Okuno et al. 2013; Fujiwara et al. 2021; Arima et al. 2022). The study revealed that even when IPM/CS was injected from the proximal point of the branches feeding the target site, no complications associated with IPM/CS embolization occurred. This finding supports the safety of IPM/CS as an embolic agent. In our study, no ischemic complications were observed, and no bleeding episodes or other complications were reported during the follow-up period.

Other microparticles, such as polyvinyl alcohol (PVA) and microspheres, can be used for embolization in LGIB cases. However, these agents are permanent embolic material with larger particle sizes than IPM/CS. Although some previous studies have shown successful embolization using PVA and microspheres introduced from the proximal site for gastrointestinal bleeding (Loffroy and Guiu 2009; Shi et al. 2017), the risk of causing an ischemic event has been demonstrated in another report (Funaki et al. 2001b; Guy et al. 1992). In addition, to prevent necrosis of bowel ischemia, the microspheres should be > 700 μm (Martí et al. 2012) which would result in proximal embolization, and there is concern about rebleeding. Regarding its particle size and permanent nature, IPM/CS might be a safer and more suitable embolic material for LGIB cases.

However, IPM/CS has some disadvantages as an embolization agent, including a lack of insurance coverage in Japan and a transient embolic effect that may lead to rebleeding. IPM/CS may be effective in occluding small blood vessels; however, it has a transient effect, indicating that it is only effective for a limited period (< 48 h). This may increase the risk of rebleeding because the blood vessel may re-open after IPM/CS has been removed. If superselective catheterization can be achieved, embolization using microcoils or other conventional embolic materials may be preferred because they have a longer-lasting effect. However, if superselective embolization is impossible, IPM/CS is a viable option for stopping the bleeding in cases of acute LGIB.

This study had some limitations, including its retrospective design and small sample size. In addition, only IPM/CS was used as the embolic agent in this study; therefore, comparing the results with those obtained using other embolic materials is impossible. The possibility that colonoscopy after TAE was not routinely performed might have resulted in some ischemic complications being undetected if they did not cause clinical symptoms. These limitations may affect the reliability and generalizability of this study's findings.

## Conclusion

This study revealed that using IPM/CS as an embolic material in TAE for acute LGIB may be safe and effective, even in cases of active bleeding.

## Data Availability

The datasets used and/or analyzed during the current study are available from the corresponding author on reasonable request.

## Abbreviations

CT: Computed tomography
Fr: French
IMA: Inferior mesenteric artery
IPM/CS: Imipenem/cilastatin
LGIB: Lower gastrointestinal bleeding
NBCA: N-butyl cyanoacrylate
SMA: Superior mesenteric artery
TAE: Transcatheter arterial embolization
